# Using hospitalization data for injury surveillance in agriculture, forestry and fishing: a crosswalk between ICD10CM external cause of injury coding and The Occupational Injury and Illness Classification System

**DOI:** 10.1186/s40621-021-00300-6

**Published:** 2021-02-15

**Authors:** Erika Scott, Liane Hirabayashi, Judy Graham, Nicole Krupa, Paul Jenkins

**Affiliations:** 1 Northeast Center for Occupational Health and Safety in Agriculture, Forestry, and Fishing, Bassett Medical Center, One Atwell Road, Cooperstown, NY 13326 USA; 2 Bassett Research Institute, Bassett Medical Center, Cooperstown, NY USA

**Keywords:** Hospitalization data, E-code, OIICS, Occupational injury, Agriculture, Forestry, Fishing

## Abstract

**Background:**

While statistics related to occupational injuries exist at state and national levels, there are notable difficulties with using these to understand non-fatal injuries trends in agriculture, forestry, and commercial fishing. This paper describes the development and testing of a crosswalk between ICD-10-CM external cause of injury codes (E-codes) for agriculture, forestry, and fishing (AFF) and the Occupational Injury and Illness Classification System (OIICS). By using this crosswalk, researchers can efficiently process hospitalization data and quickly assemble relevant cases of AFF injuries useful for epidemiological tracking.

**Methods:**

All 6810 ICD-10-CM E- codes were double-reviewed and tagged for AFF- relatedness. Those related to AFF were then coded into a crosswalk to OIICS. The crosswalk was tested on hospital data (inpatient, outpatient, and emergency department) from New York, Massachusetts, and Vermont using SAS9.3. Injury records were characterized by type of event, source of injury, and by general demographics using descriptive epidemiology.

**Results:**

Of the 6810 E-codes available in the ICD-10-CM scheme, 263 different E-codes were ultimately classified as 1 = true case, 2 = traumatic/acute and suspected AFF, or 3 = AFF and suspected traumatic/acute. The crosswalk mapping identified 9969 patient records either confirmed to be or suspected to be an AFF injury out of a total of 38,412,241 records in the datasets, combined. Of these, 963 were true cases of agricultural injury. The remaining 9006 were suspected AFF cases, where the E-code was not specific enough to assign certainty to the record’s work-relatedness. For the true agricultural cases, the most frequent combinations presented were contact with agricultural/garden equipment (301), non-roadway incident involving off-road vehicle (222), and struck by cow or other bovine (150). For suspected agricultural cases, the majority (68.2%) represent animal-related injuries.

**Conclusions:**

The crosswalk provides a reproducible, low-cost, rapid means to identify and code AFF injuries from hospital data. The use of this crosswalk is best suited to identifying true agricultural cases; however, capturing suspected cases of agriculture, forestry, and fishing injury also provides valuable data.

**Supplementary Information:**

The online version contains supplementary material available at 10.1186/s40621-021-00300-6.

## Background

Since the inception of the Occupational Safety and Health Act of 1970 (Occupational Safety and Health Act of 1970, 1970), the rates of occupational morbidity and mortality have declined in many industries. However, taken together, the agriculture, forestry, and fishing (AFF) industry sector has persistently higher rates of fatal occupational injury, when compared to the all-worker fatality rate. (Civilian occupations with high fatal work injury rates, 2018, 2019) The injury pyramid concept demonstrates that for every fatal injury event, there are many serious non-fatal injuries requiring emergency medical care, and beyond that, many injuries that require first-aid care. (WHO, 2015) While statistics related to occupational injuries exist at state and national levels, there are notable difficulties using these to understand non-fatal injuries trends in the AFF sector. In the United States, the primary source of non-fatal injury and illness data, the Survey of Occupational Injuries and Illnesses (SOII), excludes agricultural operations with less than 11 employees from being included in the sampling frame. (BLS, 2015a) Further, the United States Coast Guard has jurisdiction over commercial fishing injury through the Jones Act, as OSHA’s enforcement ends at the coastline. (United States Code, 1958) Lastly, though forestry and logging operations are within scope for the SOII, research has shown injuries and illnesses to be underestimated, as well. (Scott et al., 2020)

The goal of injury surveillance is to systematically collect, analyze, interpret and disseminate injury data, for the purpose of improving public health. (CDC, 2001) Reporting exists for a wide-variety of public health injury events, using such means as the National Hospital Discharge Survey, National Hospital Ambulatory Medical Care Survey, Emergency Department Visit Data, National Health Interview Survey, and the Behavioral Risk Factor Surveillance System, especially their Industry and Occupation (I&O) module. (Min et al., 2019; CDC, 2015a; CDC, 2015b) With the exception of the I&O module, it is very difficult to discern work-relatedness in such surveillance systems. For many years, epidemiologists have advocated to have industry and occupation routinely cataloged in the electronic health record but as of this writing, it is still not a nationally required variable. (Schmitz & Forst, 2016) The National Academies of Sciences, Engineering, and Medicine (NASEM) made this important point in a recent review focused on improving occupational injury surveillance in the twenty-first century. (National Academies of Sciences E et al., 2018)

With occupational variables missing from many injury surveillance systems, creativity is required to identify AFF injuries. Improving surveillance systems for these industries is an important goal of the National Occupational Research Agenda. (NORA Agriculture F & Fishing Sector Council, 2018) Likewise, the National Institute for Occupational Safety and Health (NIOSH) emphasized the importance of surveillance in their burden-need-impact framework for occupational injury. (Felknor et al., 2019) Such data are critical to the foundation of the public health model, serving to inform researchers and policymakers on the best use of limited research funding, apportionment for ongoing surveillance, and injury prevention efforts.

Several data sources have proved useful in identifying non-fatal injuries in agriculture, forestry and fishing. Some systems utilize electronic news clippings, which provide good detail on the type of event and source of injury for newsworthy injury events. (Weichelt et al., 2019; Weichelt et al., 2018; New-Aaron et al., 2019) The events captured via news clippings tend to be deaths or significant traumatic injury. Specialized agricultural injury surveys still exist, such as the Farm and Ranch Injury and Illness Survey (Rautiainen et al., 2019), but many national agricultural injury surveys have been discontinued due to unsustainable cost. (CDC, 2020) There is evidence that existing administrative databases, such as workers’ compensation (Missikpode et al., 2019; Kaustell et al., 2019), hospitalization data (Kica & Rosenman, 2020; Zagel et al., 2019; Scott et al., 2015; Allen et al., 2015; Grandizio et al., 2015), trauma registries (Reece et al., 2018; Grandizio et al., 2018), and pre-hospital care reports (PCR) (Scott et al., 2019; Scott et al., 2017a; Scott et al., 2011; Earle-Richardson et al., 2011; Forst & Erskine, 2009), are useful as a data source for AFF injury surveillance. While researchers are constrained to the variables contained within these systems, these datasets are often no- or low-cost, and are continually gathered, making them ideal for ongoing surveillance.

Our previous research has shown that, in a surveillance system built for the Northeast US, hospital data are an important companion to PCRs, as minimal overlap in patient records exists between the data sources. (Scott et al., 2017a) Even more, in 2015, the United States upgraded hospital data coding to the tenth version of the International Classification of Diseases – Clinical Modification (ICD-10-CM), vastly improving options for describing injuries using the expanded E-codes. (CDC, 2015c) The transition from the ninth clinical modification of ICD to the tenth saw the addition of many injury options in the external causes of morbidity codes between V00-Y99. (Hedegaard et al., 2016)

When multiple sources are used for a surveillance system, data need to be coded in a consistent manner that allows the records to be aggregated and compared in a meaningful way. One frequently used scheme in occupational health and safety (Murphy et al., 2019; Scott, 2016; Gorucu et al., 2015a; Wuellner & Bonauto, 2014; Sears et al., 2013) is the Bureau of Labor Statistics Occupational Injury and Illness Classification System (OIICS). (BLS, 2015b) The OIICS scheme is comprised of four components, each with hierarchical structures: 1) nature of the injury or illness, 2) event or exposure, 3) source of the injury or illness, and 4) the part of the body affected. Marsh and Jackson (Marsh & Jackson, 2013) suggested that there would be a benefit in having a crosswalk that could easily be used to map OIICS and ICD. However, while the Centers for Disease Control and Prevention (CDC) National Center for Injury Prevention and Control along with the National Center for Health Statistics published a report proposing a framework to present injury data using ICD-10-CM, they did not discuss OIICS. (Hedegaard et al., 2016) Creating a way to map codes between ICD-10-CM and OIICS has tremendous value for occupational health surveillance allowing for data to be merged.

This paper describes the development and testing of a crosswalk between ICD-10-CM E-codes and OIICS for AFF. By using this crosswalk, researchers can efficiently process hospitalization data and quickly assemble relevant cases of AFF injuries useful for epidemiological tracking. Doing so will improve the timeliness of existing surveillance systems, as this system not only identifies true and potential cases, but automatically codes the type of event, source of injury, and industry.

## Methods

### Development of the crosswalk

All 6810 ICD-10-CM external cause of injury codes (V0001XA to Y999) were imported into a Microsoft Access database, specially built for this review. Every code was reviewed by two occupational health and safety specialists for their AFF relatedness. These specialists have extensive training in occupational surveillance methodologies and have experience coding thousands of agriculture, forestry, and fishing-related injury records from health datasets. Previous research has demonstrated a high level of coder interrater reliability. (Scott, 2016) Each coder independently assigned a yes, no, or unsure to each ICD-10-CM E-code. The general metrics for these decisions were based on established definitions used for AFF injury surveillance at our center. Once this initial review was completed, the coders discussed any discrepancies between their choices and decided on a final determination. If the discrepancy could not be resolved, it was brought to the entire research team for review. In a final step, a lead reviewer (Principal Investigator) evaluated the 6810 records and verified the final choices made by the coding duo, making changes as necessary. In addition, the lead reviewer assigned one of nine choices to each ICD-10-CM E-code (Table 1), which determined the specific AFF industry and the degree of confidence in assigning work-relatedness (A. true case, B. traumatic/acute - industry suspected, and C. suspected traumatic/acute – industry known). For example, ICD-10-CM E-code ‘V840XXA - Driver of special agricultural vehicle injured in traffic accident, initial encounter’ would be indicative of a ‘true case’. Driving specialized agricultural equipment is typically viewed as an occupational task. Conversely, the E-code ‘W5532XA - Struck by other hoof stock, initial encounter’ would be coded as a ‘suspected case’ for the fact that we cannot be certain that the inflicted injury occurred due to a work-related event.

**Table 1:** Coding Choices for Case Determination

Case Determination	Sector
0: not a case	N/A
1: true case, Agriculture	Agriculture
1: true case, Forestry	Forestry
1: true case, Fishing	Fishing
2: traumatic/acute, suspected Agriculture	Agriculture
2: traumatic/acute, suspected Forestry	Forestry
2: traumatic/acute, suspected Fishing	Fishing
3: Agriculture, suspected traumatic/acute	Agriculture
3: Forestry, suspected traumatic/acute	Forestry
3: Fishing, suspected traumatic/acute	Fishing

After ICD-10-CM E-codes were identified and vetted, OIICS scheme was applied to them. This process was also completed by two reviewers: one primary coder and one lead coder. While the OIICS scheme contains four parts—type of event, source(s) of injury, part of body, and nature of injury—we focused on using only the type of event and source(s) of injury in applying OIICS to the ICD-10-CM E-codes. The nature of the injury and part of body information is understood directly from ICD-10-CM diagnostic codes, and therefore not necessary to recode. In addition, the type of event and source of injury codes are most critical for injury prevention activities.

### Testing the crosswalk on hospital data

The statistical software SAS 9.3 (Cary, NC) was used to complete the analyses using hospital data from three states: Massachusetts, Vermont, and New York (from 2016 and/or 2017). Hospital data included emergency department, outpatient, and inpatient records. New York data combined emergency department and outpatient records into a single undistinguishable file. There was no overlap for an individual patient visit in the state’s database for a given year. While the three levels represent various levels of patient care and severity, all are coded in ICD-10-CM and it was valuable to test the crosswalk in each type of data. These states and years of data were used because they were the most recent ICD-10-CM coded data the research team had data use approval for at the time of the analysis. Data from the ICD-10-CM E-codes identified were written into a SAS program that scanned and flagged each hospital record containing an appropriate ICD-10-CM E-code. The program appended the appropriate OIICS codes to the hospital record.

Any hospital records that contained two or more ICD-10-CM E-codes of interest were set aside for additional review. Combinations of ICD-10-CM injury codes sometimes changed the industry or the degree of confidence in work-relatedness. Analysis of the records identified as in scope were completed in Microsoft Excel (Professional Plus, 2016, Redmond, WA) using pivot tables, along with the summation, mean, minimum, and maximum functions. Since the Vermont data presented age as a range (Sears et al., 2013; BLS, 2015b; Marsh & Jackson, 2013; Leigh et al., 2014; OSHA, 2015), the analysis used the median age imputed from this range, e.g., 42 to calculate average age. Cases were not restricted to age since youth workers are not uncommon in agriculture.

All protocols were approved by the Institutional Review Board of the Mary Imogene Bassett Hospital (Bassett Medical Center).

## Results

### Development of the crosswalk

Of the 6810 E-codes available in the ICD-10-CM scheme, 263 were ultimately determined to be 1, 2, or 3 (true case, traumatic/acute, suspected AFF, or AFF and suspected traumatic/acute). Table 2 shows the results of the individual E-codes mapped to the AFF industries. Table 3 shows the results of mapping combination E-codes (more than one E-code in a given hospital record) to the AFF industries.

**Table 2:** Single ICD-10-CM E-Codes Mapped to AFF Industry

*Industry*	*Case Determination*	*Number of Single E-Codes*
Agriculture	True Case	49
Agriculture	Traumatic/Acute, Suspected Agriculture	139
Forestry	Traumatic/Acute, Suspected Forestry	1
Fishing	Traumatic/Acute, Suspected Fishing	74

**Table 3:** Combination ICD-10-CM E-Codes Mapped to AFF Industry

*Industry*	*Case Determination*	*Number of E-Code Combinations*
Agriculture	True Case	79
Agriculture	Traumatic/Acute, Suspected Agriculture	52
Fishing	Traumatic/Acute, Suspected Fishing	9

### Testing the crosswalk on hospital data

The targeted ICD-10-CM E-codes identified 9969 patient records that potentially contained an AFF injury out of a total of 38,412,241 records in the dataset. The categorization of these records can be seen in Table 4. Of these, 963 are ultimately confirmed to be true agricultural injuries. The remaining 9006 are termed suspected cases of AFF injury, meaning that the E-code was not specific enough to assign certainty to the work-relatedness. It was not possible to make any confirmed determinations of a true case of forestry or fishing from these E-codes alone.

**Table 4:** Records Identified Through ICD-10-CM E-codes Mapped to OIICS

State	Massachusetts		Vermont	New York	
Year	2016	2017	2017	2016	**Total**
**True Agriculture Records**	**128**	**87**	**93**	**655**	**963**
*Emergency Department*	109	73	80	556	
*Outpatient*	4	3	9		
*Inpatient*	15	11	4	99	
**Suspected Agriculture Records**	**958**	**849**	**506**	**3536**	**5849**
*Emergency Department*	890	794	446	3300	
*Outpatient*	8	8	55		
*Inpatient*	60	47	5	236	
**Suspected Forestry Records**	**199**	**199**	**132**	**717**	**1247**
*Emergency Department*	175	181	123	644	
*Outpatient*	3	6	8		
*Inpatient*	21	12	1	73	
**Suspected Fishing Records**	**405**	**360**	**42**	**1103**	**1910**
*Emergency Department*	380	336	33	988	
*Outpatient*	5	4	9		
*Inpatient*	20	20	0	115	
***State Totals***	1690	1495	773	6011	

* In New York emergency department and outpatient records are combined

### Descriptive epidemiology of identified patient records

Table 5 highlights the type of event and source of injury combinations that were identified for each group of cases (true agricultural cases, suspected agricultural cases, suspected fishing cases, and suspected forestry cases). For true agricultural cases, the most frequent combinations presented were contact with agricultural/garden equipment (301), non-roadway incident involving off-road vehicle (222), and struck by cow or other bovine (150). For suspected agricultural cases, the majority (68.2%) represent animal-related injuries. The majority (87.3%) of suspected fishing cases could not be classified to the type of event and source of injury. Further, all 1247 suspected forestry cases (100%) did not have identifiable types of events or sources of injury, but instead were identified due to the forestry incident location E-code. The percentage of men involved in these incidents were highest for true agricultural cases (84%), and lowest for suspected agricultural cases (44%). The average age was the lowest for true agricultural cases at 46.

**Table 5:** Type of Event and Source of Injury, Vermont, Massachusetts, & New York

Case Determination By Type of Event and Source of Injury	Count
**1: True case, Agriculture**	**963**
130 - Animal and insect related incidents, unspecified	**62**
5153 - Cattle and other bovines	59
5157 - Swine and other porcines	3
1313 - Other animal bites, nonvenomous	**11**
5150 - Mammals, unspecified	3
5157 - Swine and other porcines	8
1320 - Struck by animal, unspecified	**163**
5150 - Mammals, unspecified	10
5153 - Cattle and other bovines	150
5157 - Swine and other porcines	3
1329 - Struck by animal, n.e.c.	**1**
5159 - Mammals, n.e.c.	1
139 - Animal and insect related incidents, n.e.c.	**8**
5150 - Mammals, unspecified	8
258 - Fall on water vehicle	**3**
832 - Commercial fishing vessel	3
260 - Roadway incident involving motorized land vehicle, unspecified	**44**
869 - Off-road or industrial vehicle--powered, n.e.c	44
270 - Nonroadway incident involving motorized land vehicle, unspecified	**223**
860 - Off-road or industrial vehicle--powered, unspecified	1
869 - Off-road or industrial vehicle--powered, n.e.c	222
279 - Nonroadway incident involving motorized land vehicle, n.e.c.	**2**
860 - Off-road or industrial vehicle--powered, unspecified	1
869 - Off-road or industrial vehicle--powered, n.e.c	1
3199 - Agricultural and garden machinery, n.e.c.	**3**
0 - Blank	3
60 - Contact with objects and equipment, unspecified	**67**
310 - Agricultural and garden machinery, unspecified	67
621 - Struck by powered vehicle--nontransport	**6**
3112 - Combines	6
644 - Entangled in other object or equipment	**5**
8634 - Power take-off (PTO)	5
69 - Contact with objects and equipment, n.e.c.	**337**
3199 - Agricultural and garden machinery, n.e.c.	301
345 - Derricks and related equipment	4
3469 - Elevators, hoists, aerial lifts, personnel platforms--except truck-mounted, n.e.c.	6
8629 - Industrial vehicle, material hauling and transport--powered, n.e.c	26
7371 - Boarding, alighting--excluding slip, trip, fall--single episode	**28**
869 - Off-road or industrial vehicle--powered, n.e.c	28
**2: Traumatic/acute, suspected Agriculture**	**5849**
130 - Animal and insect related incidents, unspecified	**2332**
510 - Animals, unspecified	1770
5112 - Chickens	10
5115 - Turkeys	3
5150 - Mammals, unspecified	10
5153 - Cattle and other bovines	111
5154 - Horses and other equines	420
5157 - Swine and other porcines	8
1313 - Other animal bites, nonvenomous	**246**
5112 - Chickens	22
5113 - Ducks	2
5114 - Geese	1
5115 - Turkeys	1
5150 - Mammals, unspecified	10
5153 - Cattle and other bovines	3
5154 - Horses and other equines	147
5157 - Swine and other porcines	60
1320 - Struck by animal, unspecified	**1059**
5112 - Chickens	11
5113 - Ducks	1
5114 - Geese	2
5115 - Turkeys	1
5150 - Mammals, unspecified	57
5153 - Cattle and other bovines	225
5154 - Horses and other equines	742
5157 - Swine and other porcines	20
1329 - Struck by animal, n.e.c.	**136**
5112 - Chickens	1
5154 - Horses and other equines	135
1381 - Bitten and struck by animal	**1**
5154 - Horses and other equines	1
139 - Animal and insect related incidents, n.e.c.	**162**
510 - Animals, unspecified	141
515 - Mammals, except humans	3
5150 - Mammals, unspecified	18
2310 - Animal transportation incident, unspecified	**2**
850 - Animal- or human-powered vehicle, unspecified	2
2314 - Thrown, tipped, or fell from animal-drawn vehicle	**9**
850 - Animal- or human-powered vehicle, unspecified	9
2319 - Animal transportation incident, n.e.c.	**33**
50 - Persons, plants, animals, and minerals, unspecified	1
850 - Animal- or human-powered vehicle, unspecified	32
259 - Water vehicle incident, n.e.c.	**1**
832 - Commercial fishing vessel	1
510 - Animals, unspecified	**1**
0 - Blank	1
5154 - Horses and other equines	**5**
0 - Blank	5
5157 - Swine and other porcines	**1**
0 - Blank	1
9999 - Nonclassifiable	**1861**
0 - Blank	1
9999 - Nonclassifiable	1860
**2: Traumatic/acute, suspected Fishing**	**1910**
130 - Animal and insect related incidents, unspecified	**38**
512 - Fish, shellfish	38
1320 - Struck by animal, unspecified	**26**
512 - Fish, shellfish	26
250 - Water vehicle incident, unspecified	**10**
832 - Commercial fishing vessel	10
2521 - Collision between water vehicles	**3**
832 - Commercial fishing vessel	3
2522 - Collision between water vehicle and object	**1**
832 - Commercial fishing vessel	1
253 - Explosion or fire on water vehicle	**2**
832 - Commercial fishing vessel	2
254 - Capsized or sinking water vehicle	**3**
832 - Commercial fishing vessel	3
256 - Fall or jump from water vehicle	**1**
832 - Commercial fishing vessel	1
257 - Machinery or equipment incident on water vehicle	**1**
832 - Commercial fishing vessel	1
258 - Fall on water vehicle	**16**
832 - Commercial fishing vessel	16
259 - Water vehicle incident, n.e.c.	**5**
832 - Commercial fishing vessel	5
430 - Fall to lower level, unspecified	**135**
6691 - Piers, wharfs	135
50 - Exposure to harmful substances or environments, unspecified	**1**
9999 - Nonclassifiable	1
9999 - Nonclassifiable	**1668**
0 - Blank	4
6691 - Piers, wharfs	1
9999 - Nonclassifiable	1663
**2: Traumatic/acute, suspected Forestry**	**1247**
9999 - Nonclassifiable	**1247**
0 - Blank	6
9999 - Nonclassifiable	1241
**Grand Total**	**9969**

Note: The numeric codes are the corresponding OIICS event and source codes

## Discussion

### Development of the crosswalk

The expansion of E-codes in the ICD-10-CM framework has vastly improved our ability to use this system for AFF injury surveillance. While the list of 263 E-codes identified as AFF-related is currently all-inclusive, the possibility exists that new combinations of these 263, within a patient’s record, may permit the determination of additional true cases in future datasets. As future data are processed, the necessity to review new combinations of E-codes will drop, as a progressively larger number of these combinations are adjudicated over time. Therefore, the individual (Table 2) and combination (Table 3) E-code crosswalk will remain beneficial until ICD-11-CM is widely adopted.

### Testing the crosswalk

The crosswalk process worked equally well among the data sets from the various states; however, we acknowledge that the completeness of E-codes may vary by state. Nearly one thousand true agricultural cases have been identified using this system, which previously may have gone unknown to injury researchers, given that national sources of occupational injury data, such as SOII, are known to undercount agricultural injuries. (Leigh et al., 2014) This is especially true in the Northeast where many of the farms are small have fewer than eleven employees. (OSHA, 2015; Census of Agriculture, 2017)

Given the fact that is currently not possible to identify true cases in either forestry or fishing, the current system must be viewed as most useful for agriculture. However, E-code combinations that are identified from future hospital datasets may result in more crosswalk matches that point to true forestry or commercial fishing injuries. An example could be a patient record with ocean as the location, fishing boat as the source, and activity for civilian income leading to a case determination of ‘true fishing case’.

While the workforce in agriculture, forestry and fishing differs across the country in terms of numbers, workplace organization and technology, a strength of this approach is that it can be adopted and implemented in other regions. Over time the collective findings become more valuable as they increase in robustness.

### Descriptive epidemiology

Consistent with other agricultural injury surveillance research, events involving machinery and animals were most frequently identified in the hospital data. (Landsteiner et al., 2015; Karttunen & Rautiainen, 2013; Gorucu et al., 2015b) This emphasizes that these are important areas for immediate injury prevention attention. Similarly, the large number of suspected agricultural cases involving horses calls for increased injury prevention work, be it aimed at work-related causes or recreational riding. As with much of agriculture, there are ambiguous areas where it may be difficult to assign work-relatedness; however, it is clear that an agricultural source, in this case a horse, is the cause of a disproportionate number of injury events. This is also the case with events common in agriculture injury reporting, such as falls or overexertion. (Browning et al., 1998; Wang et al., 2011) While many falls may be occupationally related, in our approach they were classified as suspected, since there is often not enough detail in a hospital record to know the purpose of the task that lead to the injury.

The average age and gender breakdown are also consistent with previous literature. We note that the percentage of male workers is lower among suspected agricultural cases than for the other categories. In this analysis, we suspect that these are due to the relative number of horse-riding incidents, where women are more frequently injured than men. (Scott et al., 2017b)

### Limitations

E-codes are not required for hospital reimbursement; therefore, less emphasis may be placed on the completion of these codes compared to codes that relate to payment. This crosswalk will only identify and categorize injury events where hospital medical care was delivered. Therefore, injuries requiring only first aid, ambulance care with refused transport, or occupational fatalities (without hospital transport) will be missed by this system. Ideally, techniques such as this one would be used in addition to other occupational injury surveillance methods, such as text search of PCRs, news clipping services, occupational injury surveys, and existing state and federal systems such as SOII or the Census of Fatal Occupational Injury.

In addition to using other sources of data to compliment hospital data, more research needs to be done to assess if E-codes are applied at the same rate to inpatient, outpatient, and emergency department records. If the application of E-code varies, this crosswalk approach may distort the true injury trends.

Using hospital data alone, we cannot definitively assign occupational status to the suspected cases nor can we always determine the exact type of event or source of injury. Inherently, the cases are captured because of the activity at the time of the injury, not because of occupation or industry information. Therefore, it is possible that injuries occurring in a farm setting, but not related to work, would be captured. However, future research should assess the E-codes found in suspected cases compared to the information in a linked ambulance record, for example.

## Conclusions

The crosswalk provides a reproducible, low-cost, and rapid means to identify and code AFF injuries from hospital data. The use of this crosswalk is best suited to identifying true agricultural cases; however, capturing suspected AFF injury cases provides valuable data, as well. This system can be used on many state’s datasets and will remain useful until ICD-11-CM is widely adopted. One major benefit of the crosswalk, and using hospitalization data in general, is that we can better understand the direct costs of injury in these industries. Likewise, enough demographic variables exist in these datasets, as well, to target appropriate groups for public health interventions. This crosswalk represents a promising addition to the injury epidemiologist’s toolbox of surveillance techniques.

## Supplementary Information

**Additional file 1:.**

## Data Availability

The data that support the findings of this study are available from the New York Statewide Planning and Research Cooperative System (SPARCS), Massachusetts Center for Health Information and Analysis (CHIA), and the Vermont Green Mountain Care Board (GMCB) but restrictions apply to the availability of these data, which were used under license for the current study. Those interested in applying for these data may do so by contacting SPARCS [https://www.health.ny.gov/statistics/sparcs/forms/], CHIA [https://www.chiamass.gov/] and the GMCB [https://gmcboard.vermont.gov/webform/VUHDDS-PUF]. The crosswalk developed by the authors is available by a written request to the corresponding author. It will also be made publically available on our website at necenter.org.

## Abbreviations

AFF: Agriculture, forestry, and fishing
SOII: Survey of Occupational Injuries and Illnesses
I&O: Industry and Occupation
NIOSH: National Institute for Occupational Safety and Health
PCR: Pre-hospital care reports
ICD-10-CM: International Classification of Diseases – Clinical Modification tenth version
OIICS: Occupational Injury and Illness Classification System
CDC: Centers for Disease Control and Prevention
E-codes: External cause of injury codes
